# Long-term use of colchicine: “The time to guard against chronic toxicity among patients of familial Mediterranean fever”

**DOI:** 10.1186/s43166-023-00188-8

**Published:** 2023-04-03

**Authors:** Mohamed H. Emara, Abeer Hussein Abdelkader, Mohamed Said Radwan, Hassan E. Elbatae, Maysaa A. Saeed

**Affiliations:** 1 Department of Hepatology, Gastroenterology and Infectious Diseases, Faculty of Medicine, Kafrelsheikh University, Kafr El-Shikh, 33516 Egypt; 2 Department of Tropical Medicine, Faculty of Medicine, Zagazig University, Zagazig, Egypt

**Keywords:** Colchicine, Familial Mediterranean fever, Chronic toxicity, Amyloidosis, Long term, Gout

## Abstract

Colchicine is a cheap easily available and accessible drug that has been tried in different diseases which are not limited to gout, familial Mediterranean fever (FMF), Behcet’s disease, and constipation, and has recently been tried for the treatment of COVID-19 and heart diseases. There are many emerging reports of toxicity related to colchicine use. Patients with FMF are using this drug lifelong. We are sounding the alarm for monitoring patients with FMF to guard against chronic colchicine toxicity.

## To the Editor

Colchicine is a cheap easily available and accessible drug that has been tried in different diseases which are not limited to gout, familial Mediterranean fever (FMF), Behcet’s disease, and constipation [1], and has recently been tried for treatment of COVID-19 [2] and heart diseases [3].

In our oriental communities in the Mediterranean basin particularly among the Arabs, Turkish and Jewish populations the prevalence of FMF is high. Per the current practice guidelines, life-long treatment with colchicine is recommended [4]. The disease manifests during the early childhood [4]. Colchicine is given to patients with FMF with the aim to prevent FMF attacks which are the hallmark of the disease and to protect the kidney from the secondary amyloidosis that commonly complicates this disease.

We are prescribing our patients with high doses of colchicine probably for their life-long time. Furthermore, we usually assure our patients that drug-related complications would be mild and may be transient and mostly in the form of gastrointestinal tract upsets and diarrhea with no serious adverse events [4]**.** We realize the importance of caring for our FMF patients on colchicine regularly but the dilemma should tackle some points. First, should we discontinue the life-long management of FMF patients by colchicine? We believe based on the current evidence [4] that we should not stop giving colchicine to FMF patients as soon as the medicine is tolerated because the development of amyloidosis is a serious non-reversible life-threatening complication in addition to the recurrence of the disease episodes; otherwise, the introduction of interleukin-1 inhibitors is the alternative option. Second, should we monitor the therapeutic blood level of colchicine among those patients? To the best of our knowledge, there are no recommendations to check the blood level of colchicine among those patients. No therapeutic blood level check is recommended by the current guidelines [4]. Third, do we have to develop regular checkup schedules for bone marrow dysfunction, e.g., by complete blood count; neuromuscular system affection, e.g., electromyography for muscle dysfunction; and nerve conduction studies for peripheral neuropathy to check for the possible chronic toxicity of colchicine beside the routine 6-month follow-up proposed by the guidelines [4]. Furthermore, other manifestations of chronic colchicine toxicity, e.g., alopecia and electrolyte disturbances, should not be overlooked.

Hence, physicians caring for patients with FMF are asked to guide their patients on the safety profile of colchicine, monitor them at regular intervals for general evaluation, and check for manifestations of toxicity, FMF patients are at higher risk of chronic renal insufficiency and also hepatotoxicity (due to amyloidosis and other causes) which can lead to higher colchicine levels and increased toxicity. Furthermore, the literature based on the reported cases of colchicine toxicity [1–5], and the introduction of colchicine in the treatment of other diseases needs guidelines for monitoring its safety profile. Consequently, physicians should see FMF patients more regularly to monitor renal and hepatic function, ask about colchicine side effects, and ensure patients are taking their colchicine appropriately.

## Data Availability

NA

## Abbreviation

FMF: Familial Mediterranean fever
